# Topic Modelling and Sentiment Analysis of Tweets Related to Freedom Convoy 2022 in Canada

**DOI:** 10.3389/ijph.2022.1605241

**Published:** 2022-10-28

**Authors:** Shih-Hsio Huang, Shu-Feng Tsao, Helen Chen, Gaya Bin Noon, Lianghua Li, Yang Yang, Zahid Ahmad Butt

**Affiliations:** ^1^ School of Public Health Sciences, Faculty of Health, University of Waterloo, Waterloo, ON, Canada; ^2^ Faculty of Science, University of Waterloo, Waterloo, ON, Canada

**Keywords:** COVID-19, vaccine mandate, topic modeling, sentiment analysis, twitter data

## Abstract

**Objectives:** This study aimed to investigate public discourses and sentiments regarding the Freedom Convoy in Canada on Twitter.

**Methods:** English tweets were retrieved from Twitter API from 15 January to 14 February 2022 when the Freedom Convoy occurred. Unsupervised topic modelling and sentiment analysis were applied to identify topics and sentiments for each topic.

**Results:** Five topics resulted from the topic modelling, including convoy support, political arguments toward the current prime minister, lifting vaccine mandates, police activities, and convoy fundraising. Overall, sentiments for each topic began with more positive or negative sentiments but approached to neutral over time.

**Conclusion:** The results show that sentiments towards the Freedom Convoy generally tended to be positive. Five topics were identified from the data collected, and these topics highly correlated with the events of the convoy. Our study also demonstrated that a mixed approach of unsupervised machine learning techniques and manual validation could generate timely evidence.

## Introduction

It has been well-documented that people worldwide have long suffered from restrictions to contain the coronavirus disease 2019 (COVID-19) pandemic [1, 2]. As new variants have kept evolving and spreading, these restrictions have stayed for over 2 years while governments have continued imposing and lifting them, leading to great uncertainties and disruptions. When the variants appeared to be less harmful, but the pandemic has remained endlessly with restrictions still in place, the “pandemic fatigue” has emerged [3, 4], in addition to long-lasting vaccine hesitancy. Compared to the United States (US), Canada in general has been more cautious and slower to lift COVID-19 restrictions, especially around its borders with the US. Besides, Canada has put more emphasis on the COVID-19 vaccinations. Since Canada and the US have numerous business activities primarily relying on trucks to ship goods across the borders, compounded with the “pandemic fatigue” and polarised vaccine hesitancy, protests have eventually erupted.

On 15 January 2022, as the COVID-19 pandemic entered its second year, the government of Canada formally mandated that all essential service providers, including truck drivers, must be vaccinated to enter the country [5]. This was a substantial departure from past policies because truck drivers had been exempted from such a mandate to facilitate timely shipments of food and supplies [6]. A day before the mandate went into effect, a GoFundMe fundraising page was created for the “Freedom Convoy 2022” to support its organisers traveling to Ottawa to protest the new mandate [7]. Despite two suspensions and ultimate removal by the platform, the fundraiser raised over $1 million Canadian dollars for protesters, besides $6.3 million United States dollars raised by Americans on the Christian fundraising platform GiveSendGo [7, 8]. The event details are summarised in Supplementary Figure S1.

Despite condemnation from the Canadian Trucking Association, by 29 January approximately 3,000 trucks and 15,000 people were protesting in Ottawa’s downtown core in front of Parliament Hill, with an accompanying blockade forming at the border between United States and Canada at Coutts, Alberta in solidarity with the main convoy [7]. The occupation of Ottawa would continue for nearly a month, during which hateful and antisemitic signs and symbols were seen amongst protesters and concerns grew over whether the rally was a national security threat [7, 9]. This was especially concerning given the apparent support for the protest from foreign politicians and members of far-right groups, including former US President Donald Trump and QAnon [10–12]. The mayor of Ottawa declared a state of emergency on 6 February, and on 14 February, the federal government invoked the Emergencies Act for the first time in Canadian history to bring the protests to an end [7, 13]. After the breaking up of crowds and a series of arrests, including the protest organizers, Canadian Prime Minister Justin Trudeau declared the “immediate emergency situation over” and lifted the Emergencies Act on February 23, 2022 [7, 14].

Among Canadians, reactions to the Freedom Convoy were mixed. A Leger360 survey conducted in the midst of the protests found that nearly two-thirds of Canadians opposed the convoy [15]. However, an IPSOS poll found that just under half of Canadians could at least partially sympathize with the frustrations of the protesters [16]. These polls suggested a need to consider the realities faced by truckers leading up to Freedom Convoy. Compared to pre-pandemic, working conditions of truck drivers during the pandemic were more challenging since many truckers had difficulty finding places to eat, accessing restrooms, and locating parking spots [17, 18]. Furthermore, not only have truck shipment volumes been more volatile during the pandemic, but the constant movement of truckers and their interactions with other essential workers exposed them to greater risk of infection far from home and their families [19, 20]. Besides, many truckers did not consider COVID-19 as a serious health concern, so they were less likely to employ health and safety practices to protect themselves from infection [21]. During the Freedom Convoy, Twitter was utilized heavily to communicate plans and to gather and show support for protesters worldwide [9–13, 22–24]. Twitter has been used extensively by researchers throughout the pandemic to examine the public discourses and sentiments surrounding COVID-19 and responses to government policies [25–28]. Such studies have employed machine learning (ML) techniques for topic modelling and sentiment analysis.

Topic modelling involves natural language processing (NLP) that allows researchers to analyze text data to determine word patterns [29]. Sentiment analysis is another ML method used to analyze text data according to metrics associated with emotions, opinions, or attitudes [30]. One metric classifies text into “positive,” “negative,” or “neutral” [30] based on the polarity of the sentiment. Another metric assigns a sentiment score, referred to as valence [31], to the text based on a spectrum, such as a number between −1 and +1, where −1 represents negative valence, and +1 represents positive valence. By knowing the sentiment of a tweet, and the sentiment of topics identified among the tweets, it offers better interpretability [29–31]. By combining sentiment analysis with topic modelling, this offers improved interpretability towards how Twitter users feel towards the Freedom Convoy movement. Such social listening approaches (i.e., using topic modeling and sentiment analysis) have gained popularity and extensive research over the pandemic since researchers have turned to harness social media data to better understand public discourse and identify issues [25–28, 32–34]. Unsupervised Latent Dirichlet Allocation (LDA) topic modelling [29, 35] and the Valence Aware Dictionary and sEntiment Reasoner (VADER) [30, 36] have been commonly used in various social listening or health infodemiological studies [32–34]. They have consistently provided reasonable results, although they are not the most advanced techniques [35, 36].

Given the relative recency of the Freedom Convoy, there is currently a dearth of publications on this topic. Analyzing users’ discourses and sentiments towards the Freedom Convoy may provide insights for decision makers knowing how to address similar events happen in the future. In addition, understanding public opinions will allow governments to efficiently develop strategies to prevent the escalation of strong opinions into action.

Therefore, the objective of this study was to apply an unsupervised LDA topic modelling and VADER sentiment analysis to investigate and understand public opinions toward Canadian Freedom Convoy on Twitter. The primary hypothesis of this study was that Twitter could be used to provide a summary of sentiments towards the Freedom Convoy 2022 movement and their relative popularity. The key research questions of this study were (i) what were the sentiments of Twitter users towards the Freedom Convoy 2022 movement, by topic, based on English tweets between 15 January and 14 February 2022, and (ii) how do topics and sentiment towards the Freedom Convoy 2022 movement compare based on likes and retweets?

## Methods

### Study Design

This study is an ecological study using data from Twitter from 15 January to 14 February 2022, based on the Freedom Convoy activities in Canada.

### Data Collection

**FIGURE 1:**
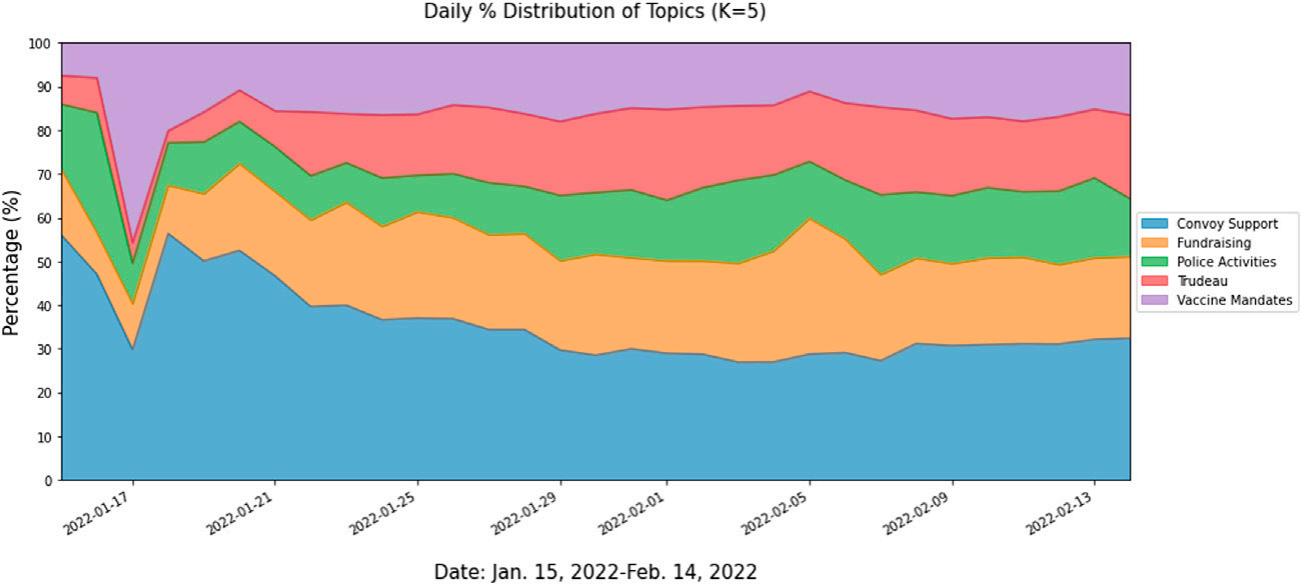
Distribution of 5 topics over time (Waterloo, Canada. 2022). Study Name: Topic Modelling and Sentiment Analysis of Tweets related to Freedom Convoy 2022 in Canada.

English tweets were collected *via* the Twitter application programing interface (API) from 15 January to 14 February 2022, according to the Freedom Convoy activities in Canada shown in Figure 1. 15 January 2022 was chosen because it was the official start date of the US-Canadian COVID-19 border restrictions. 14 February 2022 was chosen because it marked the day when the Canadian government enacted the Emergencies Act to end the protests. No location data was specified for collection due to limited access to accurate location data from Twitter users. Keywords and hashtags used to retrieve tweets are listed in Table 1. Initial data collection resulted in 3,742,209 tweets. Post-retweets, duplicate tweets and blank tweets removal resulted in 560,140 tweets. The overall analysis flow diagram is summarized in Supplementary Figure S2. Abbreviations or synonyms of frequent bigrams were also replaced with one base form. For example, “justin_trudeau” was replaced with “trudeau” and “freedom convoy” was replaced with a neutral word to mitigate positive biases.

**TABLE 1:** Keywords and hashtags used to retrieve tweets (Waterloo, Canada. 2022). Study Name: Topic Modelling and Sentiment Analysis of Tweets related to Freedom Convoy 2022 in Canada.

Keywords	Hashtags
freedomconvoy, freedomconvoy2022, (freedom AND convoy), (trucker AND protest), (truck AND protest), karenkonvoy, flutruxklan	#freedomconvoy, #freedomconvoy2022, #karenkonvoy, #KarenKonvoy, #flutruxklan, #FluTruxKlan

The following parameters were collected for each tweet:

- author_id: a unique ID assigned to the Twitter user account.
- create_at: date and time of tweet creation.
- id: a unique tweet ID assigned to each tweet. An account can have more than one tweet ID.
- like_count: number of likes from the tweet.
- retweet_count: number of retweets from the tweet.
- text: tweet content.

### Data Preprocessing

Following procedures descried in prior studies [25–28], for topic modelling, all tweets were transformed into lowercase and Unicode strings were transformed into American Standard Code for Information Interchange (ASCII). Non-texts were removed, including uniform resource locator (URL), punctuations, and special characters (!"#$%&'()*+, -./:;<=>?@[\] ^_`{|}∼). Additionally, all emojis, emoticons, numbers within words, numbers from sentences, and trailing spaces were removed. Repeating character sequences that are greater than three letters. For instance, “hahaha” were converted into three letter character sequences such as “hah.” Next, stop words were removed using the spaCy English stop words dictionary [37, 38]. The tweets were then lemmatized using WordNetLemmatizer and stemmed using PorterStemmer Words were then converted into phrases using Phrases from Python’s Gensim package [39], and unigrams and bigrams that occur less than 10 times within the dataset were disregarded. Tweets were then tokenized using SKLearn’s CountVectorizer [40] to generate the count of every token contained in the dataset. Terms that appeared in more than 90% of the total dataset or words that appeared less than 10 times were removed to filter out frequent and infrequent terms following existing literature [40]. Unsupervised LDA topic modelling was applied using Python’s SKLearn package [41] to generate potential keywords for identifying topics. Topic modelling was specified to search for up to 15 topics. Topic optimization was done according to coherence score, and then manually validated by all researchers reviewing the preliminary keywords for each topic generated by LDA to collaboratively interpret topics for further data cleansing. Synonyms of keywords used for data collection query that emerged throughout all topics during topic modelling were removed as additional stop words to improve interpretation of emerging topics from further topic modelling. Additional stop words are listed in Supplementary Table S1.

For sentiment analysis, emojis, emoticons, punctuations, and special characters were added back to each tweet to capture original sentiments as accurately as possible. Words were reverted back into their original forms prior to repeated length removal, stemming, lemmatizing, and phrasing. All stop words were also added back into the tweets to provide additional context for sentiment analysis. However, the capitalization of the first letter and all letters of the words were then replaced with neutral terms through conversion into a random mix of characters and integers. This was done to avoid terms used to refer to the convoy, such as “freedom convoy” or “truckers protest”, in which the terms “freedom,” or “protest,” carry sentiment weights, from biasing the overall sentiment of a tweet. VADER [42] sentiment was used to calculate the sentiment value associated with each tweet. The algorithm generates a normalized compound value between -1 and +1. A score equal to or greater than +0.05 is categorized as “positive,” a score equal to or lower than −0.05 is categorized as “negative,” and categorized as “neutral” if the score is between −0.05 and +0.05. Initial sentiment analysis was completed without taking retweets and likes into consideration. Additional sentiment analysis was conducted using retweets and likes as multiples of the original tweet +1, where +1 represents the count of the original tweet, multiplied by the sentiment score as below:

Weighting of retweets=Compound score*(Retweet Count+1)

Weighting of likes=Compound score*(Like Count+1)

As retweets were removed during the data cleaning, to calculate daily sentiment score based on retweets, the sum of the daily weighted retweets was divided by the sum of the daily retweet count +1, as an additional tweet is generated when a tweet is retweeted. Since liking a tweet does not generate an additional tweet, each tweet’s weighted sentiment score by likes was standardized through standardized scaling, and the daily sentiment scores based on likes were calculated by taking the mean of the scaled daily weighted likes.

### Manual Validation

Manual validation of topic modelling was done by researchers on 240 tweets, randomly sampled from emerging topics identified from topic modelling. Similarly, manual validation of sentiment analysis was done by all researchers on 415 tweets, consisting of random samples of positive, neutral, and negative sentiment tweets. Inter-rater agreement percentage of validation results are reported in–Supplementary Tables S2, S3.

## Results

Table 2 shows the topics given by the unsupervised LDA topic modelling. These five topics were chosen based on the research questions, i.e., public sentiment towards the Freedom Convoy movement and researchers’ interpretations with corresponding keywords from each topic’s keyword outputs generated by the unsupervised LDA topic modelling and manual validation.

**TABLE 2:** Topics, keywords, and synonyms generated from the unsupervised Latent Dirichlet Allocation (Waterloo, Canada. 2022). Study Name: Topic Modelling and Sentiment Analysis of Tweets related to Freedom Convoy 2022 in Canada.

Topic Number	Human interpretations	Top 15 emerging words	Number of tweets (% of total tweets)	Total retweets and likes
1	Support for the convoy	Support, COVID, stand, cdnpoli, govern, arrest, driver, news, call, peopl, ralli, terrorist, tyranni, time, today	172,410 (30.78%)	Retweets: 825,352
				Likes: 2,984,818
2	Political arguments toward the current Prime Minister	trudeau, like, world, video, thank, peac, honkhonk, love, look, speak, movement, share, power, lie, flag	116,931 (20.88%)	Retweets: 541,253
				Likes: 2,105,367
3	Opinions towards lifting COVID-19 vaccine mandates in Canada	mandat, protest, report, live, end, start, vaccin_mandat, want, stop, govern, vaccin, countri, way, american, ottawa	84,545 (15.09%)	Retweets: 532,782
				Likes: 1,953,956
4	Opinions towards police activities to contain the convoy	ottawa, polic, day, come, break, ontario, weekend, week, protestor, head, kid, help, citi, thousand, actual	97,723 (17.45%)	Retweets: 458,877
				Likes: 1,734,801
5	Fundraising for the convoy	peopl, right, medium, know, need, donat, think, thing, gofundm, go, want, let, fund, watch, organ	88,531 (15.81%)	Retweets: 539,073
				Likes: 1,955,348

Coherence score generally appeared to increase as the number of topics for unsupervised learning increased. As the number of topics increased, topics formed more granular clusters corresponding to specific events relating to the convoy movement, whereas less topics appeared to capture overall themes of the movement. Figure 1 demonstrate the percentage distribution of tweets identified from the five topics that emerged from unsupervised LDA topic modeling. In Figure 1, the first topic showing general support for the convoy covers about 31% of tweets throughout the study period The second topic containing politics-related keywords, such as “Trudeau,” or “government,” demonstrates heated political and polarised arguments toward the convoy and general politics. The third and fourth topics show public opinions toward lifting or imposing Canadian vaccine mandates and police activities to tackle the convoy, respectively. The last topic is especially about the convoy’s fundraising activities and promotions. Results from the unsupervised learning correspond to other findings that utilize LDA topic modelling as well [43].

**FIGURE 2:**
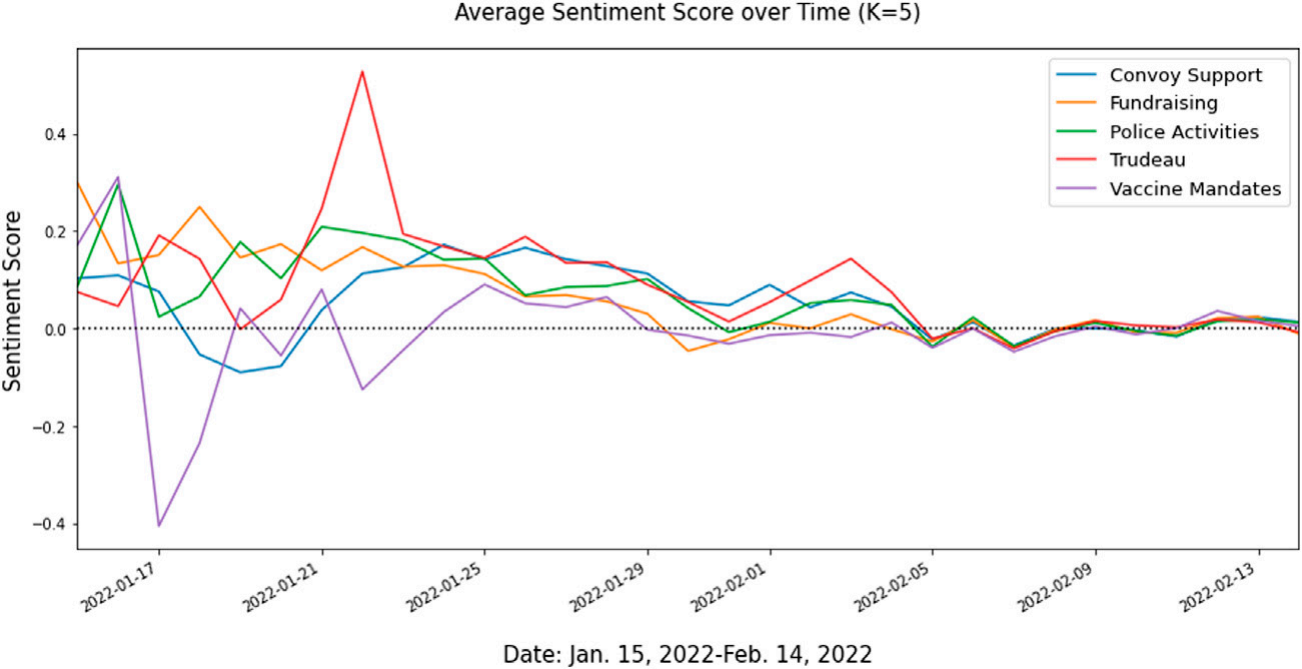
Average daily sentiment score by topic (Waterloo, Canada. 2022). Study Name: Topic Modelling and Sentiment Analysis of Tweets related to Freedom Convoy 2022 in Canada.

Figure 2 illustrates the average sentiment score for each topic over the study period. Sentiment scores generally is positive towards the movement but fade out to neutral over time, except for the third topic about the vaccine mandates with several negative sentiments in the beginning.

Overall sentiment scores when weighting by retweets versus likes differed in magnitude by much as shown in Supplementary Figures S3, S4. When the average sentiment scores were weighted by the number of retweets, it demonstrated variations in the average sentiment scores among topics, but it still aligned with the overall trend in Figure 2. However, when the average sentiment scores were weighted by the number of likes, the overall sentiment trends were more flattened than that in Figure 2, and only peaks and down peaks stood out. For example, the fourth topic’s average sentiment score had a substantial nosedive on 5 February 2022, the day before Ottawa City Mayor officially declared the state of emergency and increased police presence to contain the convoy. Twitter already reflected considerable negative discussions toward it.

Twitter defines retweet as a feature to help a user share a tweet with their followers [44]. Since retweets represent a form of sharing, this may suggest weighting the tweets by retweets may capture initial duplicate tweets that were filtered out. However, likes also represent a form of sharing. Twitter defines likes as feature to show appreciation for a tweet [45]. While a user’s likes can still be viewed by their followers, the action of liking a tweet does not repeat the original tweet to generate a new unique tweet. As a result, the similarity in sentiment magnitude of likes to retweets cannot be explained by the exclusion of duplicate tweets. This suggests that the sentiments towards certain topics can be polarized by retweets or likes and may actually drown out other sentiments within the topic. This is illustrated in Table 3, as the percentage of sentiment categories across certain topics seem similar overall, but at certain time points, the interpretation of certain topics seems to be biased towards one or the other sentiment category. This may capture a sort of bias of extreme sentiment opinions that tend to capture more attention on social media, creating an over-representation of extremes towards events that may not accurately reflect the overall picture. For example, around 16 January 2022, sentiment for the third topic “Vaccine Mandates” peaked at +0.3 and remains close to +0.6 when weighted by retweets, but close to +0.07 when weighted by likes. Thus, retweets and likes can actually change the magnitude of a topic.

**TABLE 3:** Percentage of tweets by sentiment category within each topic (Waterloo, Canada. 2022). Study Name: Topic Modelling and Sentiment Analysis of Tweets related to Freedom Convoy 2022 in Canada.

	Topics				
Sentiment category	1: Convoy Support	2: Trudeau	3: Vaccine Mandates	4: Police Activities	5: Fundraising
Negative	33.90%	37.67%	38.86%	36.04%	39.30%
Neutral	26.01%	19.75%	24.18%	25.34%	20.17%
Positive	40.09%	42.58%	36.96%	38.62%	40.53%

## Discussion

Although keywords and hashtags for both supporting and opposing the convoy were included to retrieve relevant tweets, only pro-convoy tweets have surprisingly emerged and resulted in the first topic. Anti-convoy tweets with “#karenconvoy” and “#flutruxklan” resulted in 84,389 tweets (15.1%) out of total tweets. When considering retweets, tweets containing anti-convoy words reduced down to 7.9% of total tweets and retweets [(84,389 + 212,912)/3,742,209]. We investigated further and realized that “#karenconvoy” and “#flutruxklan” did not appear on Twitter until 23 January and 24 January 2022, respectively. Compared with “freedomconvoy” already appearing on 15 January 2022, this potentially led to a smaller number of anti-convoy tweets to in our data sample. Another possible reason could be that tweets against the convoy were actually classified into other topics by LDA, instead of a separate topic showing direct opposition to the convoy. Overall, the magnitude of anti-convoy tweets was so small that it did not have a substantial impact on the overall data analysis, and thus it did not stand out as a separate topic according to the LDA topic modeling.

Sentiment scores began with positive and negative for different topics, and then all eventually approached neutral over time. This became more apparent when the numbers of retweets and likes for each tweet were taken into account. Further analysis on each topic, however, revealed that although on average sentiments approached neutral, positive and negative sentiments towards each topic stayed roughly consistent. This suggests extreme sentiments towards a topic faded out over time, but polar sentiments towards topics remained consistent. This becomes more transparent by examining sentiments after 29 January 2022, when the main rally outside Parliament Hill, as it serves as a turning point where most topics approached neutral. However, it is hard to tell the distinction between pro- and anti-convoy from positive and negative sentiments. During the manual validation, the researchers noticed that many tweets with negative sentiments reflected mostly frustrations and blame on political leadership, but there were also negative sentiments towards convoy supporters along with their actions and events of the convoy. This was true across topics, whereas positive sentiments across topics mostly reflected support for the movement. It’s likely that the extreme sentiments on social media corresponding to the events of the convoy generated more support, which exacerbated the protest from the US-Canada border COVID-19 vaccine mandate into a general protest against COVID-19 restrictions, in general. The shift in sentiment patterns prior to and post the Parliament Hill protest on 29 January 2022 suggests that a build-up of sentiments on social media could potentially act as a precursor to foreshadow events that may require early actions to mitigate consequences. Using sentiment analysis to inform decision-making once an event has already happened may be too late, as most topics post 29th January shift towards neutral, which offers limited interpretability.

However, the results from the sentiment analysis need to be considered with topics output from the topic modeling to better understand its context. In the other words, it is not helpful to consider only the results from the sentiment analysis. By listening to discourses about the Freedom Convoy on Twitter, our study has demonstrated one type of social listening approach using both topic modeling and sentiment analysis. Decision-makers, therefore, can combine social listening results from social media with insights from other data sources to have a clearer picture in order to inform their decisions during the ever-changing event like the Freedom Convoy.

Another finding during manual topic validation was that tweets with increased number of topics, keywords relating to COVID-19, such as “vaccin_mandat” or “COVID” appear, suggesting a distinct topic. The same occurs for fundraising, with terms such as “gosendgo”, “donat”, and “gofundme” appearing as keywords within a topic as topic number increases. However, most tweets that discuss COVID-19 restrictions and fundraising were mostly intertwined within tweets about the Freedom Convoy, in general. For example, tweets about fundraising mostly discussed donating to the convoy to support the movement, whereas tweets about COVID-19 vaccine mandates related to talks about freedom and supporting the convoy to remove the mandates. This may explain why there are many overlaps between daily sentiments for the five topics. By increasing the number of topics (see Supplementary Figure S5), clusters become more granular and the list of top words that correspond to each topic becomes more interpretable. However, the actual tweets themselves do not show a clear pattern relating to the interpretation from only the keywords. Pattern overlaps between daily sentiments for different topics, despite different top words from topic modelling, may suggest that different opinions towards events of the convoy may emerge as granular topics from unsupervised learning. However, this also suggests that unsupervised topic modelling offers limited interpretability. Many tweets were context-specific and required knowledge of events relating to convoy movement happening at the time in order to manually interpret the theme. Including replies and quotes in the dataset also meant that tweets directed at certain user handles, such as political leaders, required some domain knowledge, which is not provided during unsupervised topic modelling. These may explain why there is a low inter-rater agreement percentage on the topic validation.

The restrictions and vaccine mandates have been polarised since the beginning. Although public health professionals have shown scientific evidence to support the restrictions and vaccine mandates, the ideology of “Freedom” among the public and certain political leaders have made the public health recommendations not as straightforward as public health professionals would think [46–50]. Public health professionals tend to distance us from politics, but ordinary people are indeed inexplicitly or explicitly influenced by political ideologies. As prior social listening studies have demonstrated [25–28, 33, 34], topics associated with political discourse have consistently resulted from the topic modeling, including our study. Therefore, when providing or implementing public health measures or practices, public health professionals are recommended to expect and prepare for polarizations and resistance from the public. In addition, social listening studies, such as our study, can be a supplementary tool for public health professionals or decision-makers to identify issues timely and provide clearer communications thereafter.

### Limitations

The study had several limitations. Firstly, it’s possible that the keywords used for data collection are biased towards support for the movement in the beginning, although keywords and hashtags against the convoy emerged 1 week later. Besides, those who did not support the movement may not use official hashtags or terms associated with the movement as it may signify support for the movement. Instead, some may opt to use other related terms to bring attention to the movement. It’s possible that sentiment towards convoy support may be balanced out by a similar amount of positive and negative sentiments towards the end of data collection period as Figure 2 shows that the average sentiment scores moved toward neutral. Another limitation is regarding the validity of the unsupervised sentiment analysis. Although VADER generally performed generally well for classifying and scoring the sentiments for the tweets, during manual validation, we found that tweets with more swear words tended to be scored as more negative, in general, regardless of the context. The algorithm also tended to classify texts that had a lot of capitals as positive, regardless of content. This led to misclassification of some negative sentiment tweets written in capital as being classified as positive sentiment. Furthermore, as no specific keywords related to COVID-19 or fundraisers were used for data collection, it may explain why related keywords from topic modelling emerge more when topics become more granular (K = 14), versus when topics are broader (K = 5). The other possibility is that some tweets may contain more than one topic, which was something researchers noticed during manual topic validation. Thus, rather than classifying a tweet under one representative topic, further analysis by classifying tweets under multiple topics if the topic probability meets a set probability threshold may reveal more topic patterns and increase the number of interpretable topics.

### Conclusion

In conclusion, our results have shown that sentiments towards the convoy generally tended to be positive, although this may be a result of keywords used for data collection. However, we removed “freedom” during data preprocessing to mitigate the bias toward positive sentiments, but the positivity remained. Five topics were identified from the data collected, and these topics highly correlated with the events of the convoy. The emergence of negative sentiments towards the vaccine mandates may explain some of the timing behind political events that emerged during the movement’s timeline. Taking retweets and likes into account also enhances existing sentiments for a subset of opinions. As retweets and likes represent methods of illustrating opinions towards a tweet without having to add original content, taking these enhancements into account when analyzing sentiments could explain changes in polarity of sentiments. Our research also demonstrated that a mixed approach of unsupervised topic modelling and manual validation could generate timely evidence. Therefore, additional methods of social media analysis, such as network analysis, may be required to complement unsupervised results in order to support informed decision-making.
