# Supplementary material for: Topic Modelling and Sentiment Analysis of Tweets Related to Freedom Convoy 2022 in Canada
Source: Int J Public Health. 2022 Oct 28;67:1605241. doi: 10.3389/ijph.2022.1605241 (PMC9649435; doi:10.3389/ijph.2022.1605241)

**Supplementary Materials**

Table S1: Additional stopwords removed (Topic Modelling and Sentiment Analysis of Tweets related to Freedom Convoy 2022 in Canada. Waterloo, Canada. 2022)

| Additional Stopwords | |
| --- | --- |
| Freedom Convoy Keyword Synonyms | 'freedomconvoy', 'freedomconvoycanada', 'convoyforfreedom', 'truckerprotest', 'truckerconvoy', 'truckersconvoy', 'truckerforfreedom', 'truckersforfreedom', ‘karenkonvoy’, ‘karenconvoy’, ‘flutruxklan’, ‘flutrucksklan’, ‘flutrucksclan’ |
| Frequent Emerging Keywords Across Topics | 'freedom', 'convoy', 'protest', 'truck', 'trucker', 'truckers', 'canada', 'canadian', 'canadatruckers' |
| Synonymous words | ‘justin_trudeau’->’trudeau’, ‘govt’->’govern’ |

Table S2: Inter-rater agreeance for topic modeling (Topic Modelling and Sentiment Analysis of Tweets related to Freedom Convoy 2022 in Canada. Waterloo, Canada. 2022)

| Sample of matched topic tweets | 77 |
| --- | --- |
| Total sample of tweets | 240 |
| Agreeance % | 32.1% |

Table S3: Inter-rater agreeance for sentiment analysis (Topic Modelling and Sentiment Analysis of Tweets related to Freedom Convoy 2022 in Canada. Waterloo, Canada. 2022)

| Sample of matched sentiments tweets | 354 |
| --- | --- |
| Total sample of tweets | 415 |
| Agreeance % | 85.3% |

Figure S1: Event timeline (Topic Modelling and Sentiment Analysis of Tweets related to Freedom Convoy 2022 in Canada. Waterloo, Canada. 2022)


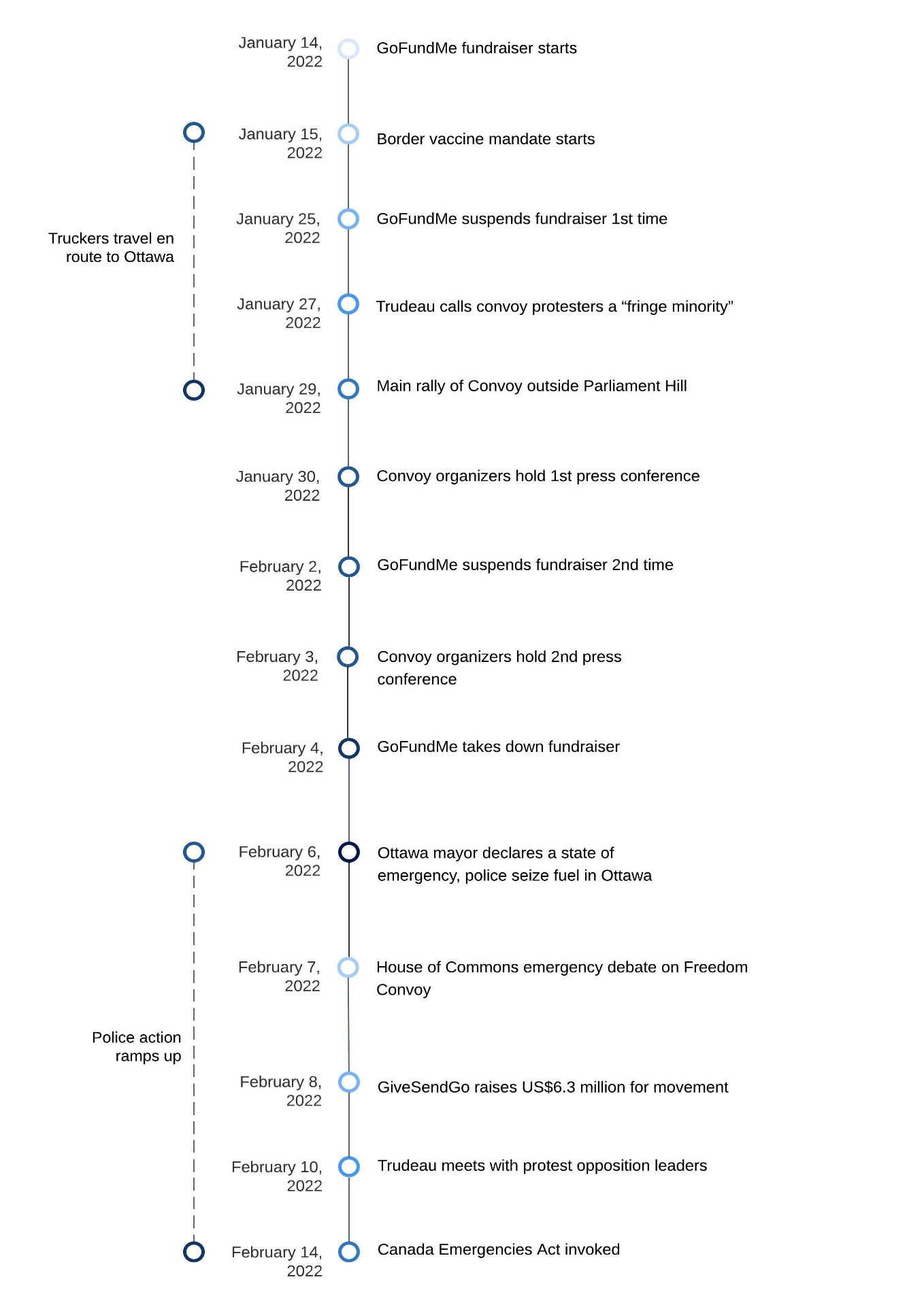


Figure S2: Analysis flow (Topic Modelling and Sentiment Analysis of Tweets related to Freedom Convoy 2022 in Canada. Waterloo, Canada. 2022)


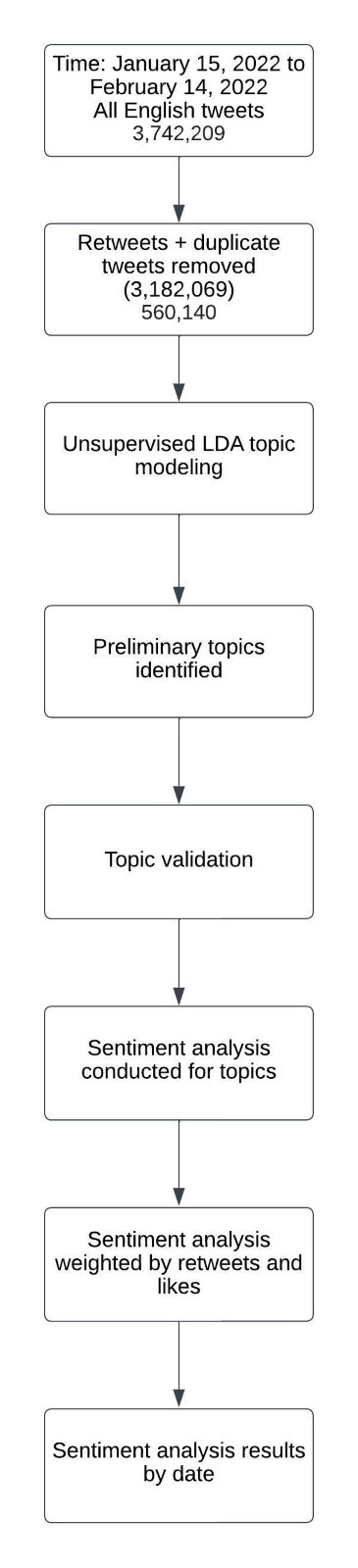


Figure S3: Average daily sentiment score by topic weighted by retweets (Topic Modelling and Sentiment Analysis of Tweets related to Freedom Convoy 2022 in Canada. Waterloo, Canada. 2022)


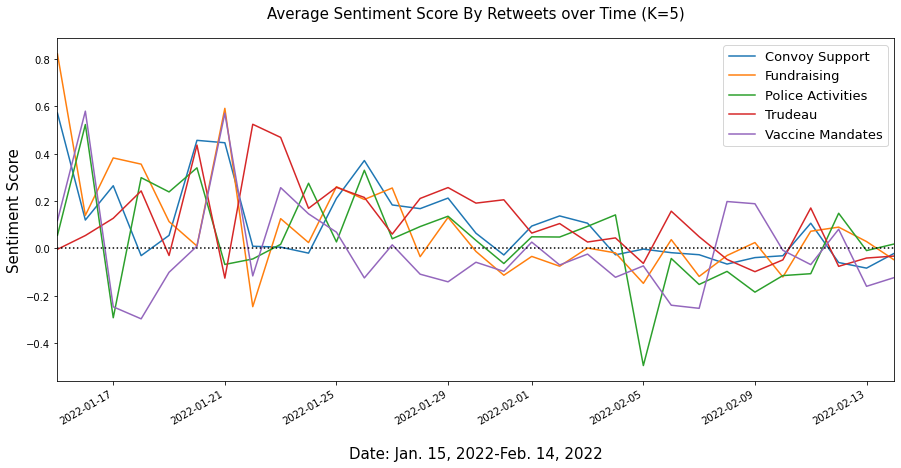


Figure S4: Average daily sentiment score by topic weighted by likes (Topic Modelling and Sentiment Analysis of Tweets related to Freedom Convoy 2022 in Canada. Waterloo, Canada. 2022)


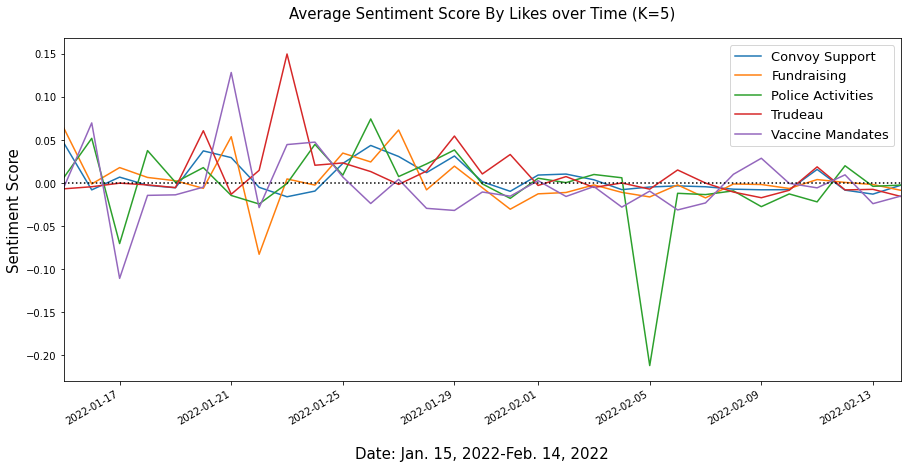


Figure S5: Distribution of 14 topics over time (Topic Modelling and Sentiment Analysis of Tweets related to Freedom Convoy 2022 in Canada. Waterloo, Canada. 2022)


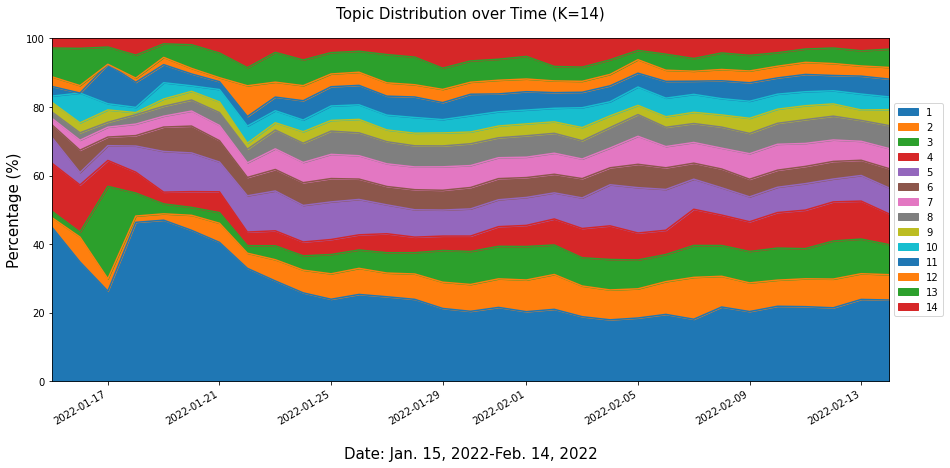

Supplement: Supplementary file 1 [file DataSheet1.docx]
